# Conceptual Analysis: A Social Neuroscience Approach to Interpersonal Interaction in the Context of Disruption and Disorganization of Attachment (NAMDA)

**DOI:** 10.3389/fpsyt.2020.517372

**Published:** 2020-12-23

**Authors:** Lars O. White, Charlotte C. Schulz, Margerete J. S. Schoett, Melanie T. Kungl, Jan Keil, Jessica L. Borelli, Pascal Vrtička

**Affiliations:** ^1^Department of Child and Adolescent Psychiatry, University of Leipzig, Leipzig, Germany; ^2^Department of Neurology, Max Planck Institute for Human Cognitive and Brain Sciences, Leipzig, Germany; ^3^Department of Psychology, University of Erlangen-Nuremberg, Erlangen, Germany; ^4^THRIVE Laboratory, Psychology and Social Behavior, University of California, Irvine, Irvine, CA, United States; ^5^Research Group “Social Stress and Family Health”, Max Planck Institute for Human Cognitive and Brain Sciences, Leipzig, Germany; ^6^Department of Psychology, Centre for Brain Science, University of Essex, Colchester, United Kingdom

**Keywords:** disorganized attachment, neglect and abuse, maltreatment, co-regulation, social interaction, social neuroscience

## Abstract

Humans are strongly dependent upon social resources for allostasis and emotion regulation. This applies especially to early childhood because humans—as an altricial species—have a prolonged period of dependency on support and input from caregivers who typically act as sources of co-regulation. Accordingly, attachment theory proposes that the history and quality of early interactions with primary caregivers shape children's internal working models of attachment. In turn, these attachment models guide behavior, initially with the set goal of maintaining proximity to caregivers but eventually paving the way to more generalized mental representations of self and others. Mounting evidence in non-clinical populations suggests that these mental representations coincide with differential patterns of neural structure, function, and connectivity in a range of brain regions previously associated with emotional and cognitive capacities. What is currently lacking, however, is an evidence-based account of how early adverse attachment-related experiences and/or the emergence of attachment disorganization impact the developing brain. While work on early childhood adversities offers important insights, we propose that how these events become biologically embedded crucially hinges on the context of the child–caregiver attachment relationships in which the events take place. Our selective review distinguishes between direct social neuroscience research on disorganized attachment and indirect maltreatment-related research, converging on aberrant functioning in neurobiological systems subserving aversion, approach, emotion regulation, and mental state processing in the wake of severe attachment disruption. To account for heterogeneity of findings, we propose two distinct neurobiological phenotypes characterized by hyper- and hypo-arousal primarily deriving from the caregiver serving either as a threatening or as an insufficient source of co-regulation, respectively.

Disturbances in childhood family functioning account for approximately a quarter to a third of youth- and adult-onset mental disorders (1, 2). Attachment theory and research offer an in-depth theoretical account of how family caregiving relationships from infancy onwards impact development, for better and for worse, across a vast array of psychosocial domains (3). Much work has attempted to leverage attachment theory to shed light on mechanisms underlying the effects of adverse early caregiving experiences on later mental health (4), with most data showing the highest risk to emanate from disorganized attachment (5–7). However, aside from a few recent pioneering empirical studies (8–14), a social neuroscience perspective encompassing disorganized and maltreatment-related disruption of attachment is still notably absent. Recently, a comprehensive functional neuro-anatomical model of human attachment was proposed [NAMA (15–17)]. NAMA describes a prototypical attachment pathway reflecting psychological processes activated in attachment-relevant situations, which is likely to be maintained by four neural modules. It further summarizes the evidence available to date on how inter-individual differences in the three major typical (or “organized”) attachment patterns coincide with anatomy and function within, and connectivity between these modules. However, the account of NAMA is notably incomplete in that disorganized attachment is largely omitted due to a paucity of data and the lack of an according conceptual social neuroscience framework. The present paper aims to begin to fill this gap. After providing a brief conceptual overview of organized and disorganized attachment, we extend NAMA to a functional neuro-anatomical model of disrupted attachment (NAMDA). To support our speculations on the putative neurobiological underpinnings of disorganized attachment, we draw on *direct* and *indirect* empirical evidence stemming from studies utilizing samples assessed for attachment disorganization and maltreatment histories, respectively.

## Organized and Disorganized Attachment in a Nutshell

Attachment theory claims that children's repeated interactions with their primary caregiver(s) shape their early organization of attachment, thereby guiding behavior in attachment-relevant situations (18–22). Following a developmental sequence, children progress from overt behavioral strategies organized at a procedural level to a later representational organization (23), referred to as internal working models of attachment (24, 25). Children whose caregivers reliably respond in a sensitive manner to their needs tend to adopt an “organized” (i.e., attachment strategy-driven) and secure attachment pattern (19, 26). Thus, they turn to their caregivers in times of distress (safe haven function) and explore in the caregiver's vicinity in times of safety (secure base function), ultimately facilitating a sense of self-efficacy and trust in others, more generally (27).

Conversely, children whose caregivers are merely inconsistently available in times of distress tend to adopt an insecure anxious–ambivalent strategy, involving hyperactivation of the attachment system during distress (e.g., excessive proximity seeking and maintaining), an organized strategy thought to maximize the amount of nurturance elicited from caregivers. In turn, offspring of caregivers who typically thwart their child's bids for contact and are relatively unresponsive to their emotional signals tend to adopt an insecure–avoidant strategy of suppressing (outward signs of) distress, an organized strategy thought to minimize the caregiving burden and odds of further rejection by caregivers (28, 29). These strategies reflect (co-)regulatory mechanisms comprising overdependence on others (anxiety) or overemphasis on self-reliance (avoidance) while they remain expedient (and thus organized), achieving the evolutionarily highly adaptive goal of maintaining sufficient proximity to the caregiver in a given environment (29, 30). Hence, they preserve (limited) co-regulation by caregivers.

By contrast, according to Main (31), disorganized attachment reflects a breakdown of the aforementioned organized strategies and occurs when the child experiences “fright without solution” within the attachment relationship [(32), p. 484]. This state is thought to emerge because the distressed child requires comfort from attachment figures (AFs) which, however, is (felt to be) largely unattainable because the AFs themselves have become associated with alarm (4). The classic case cited in this context is that of caregivers who expose their child to physical abuse so that they simultaneously represent both the primary source of comfort and the primary source of distress for their child. This circumstance is thought to give rise to conflicting motivations on behalf of the child involving co-existing tendencies to approach and avoid their frightened/frightening caregivers, eventuating in a set of apprehensive, disoriented, or contradictory behaviors (e.g., seeking comfort with markedly averted face) (33, 34). It is noteworthy, however, that akin to Ainsworth's early work, Main conceives of fear linked to the AF (e.g., due to maltreatment) as having a disorganizing influence on the child, resulting in a breakdown of organized attachment strategies, i.e., inhibiting bids for co-regulation from caregivers under distress and/or exploration in caregivers' vicinity under calm conditions. Conversely, others consider fear linked to the AF as an organizing force and “disorganization” to be a misnomer (35, 36). In line with Ainsworth's later work, for Crittenden, fear of the AF thus promotes excessive tendencies to either (1) overemphasize cognitive predictability at the expense of negative affect expression or (2) overamplify negative affect at the expense of cognitive predictability (35). While these strategies are thought to result in a lack of integration of cognition and affect, they may serve a self-preserving function, maximizing survival odds (e.g., compulsive compliance with caregivers' demands in the case of physical abuse) (37)^1^.

### Precursors and Mental Health Sequelae of Disorganized Attachment

As noted above, the state of “fright without solution” is thought to lie at the heart of disorganized attachment. However, “fright without solution” often, though by no means invariably, entails that caregivers act as a *source* of alarm for the child, as in the case of physical abuse (38). Indeed in a meta-analysis on maltreatment and disorganization, the effects of abuse and neglect on disorganization were almost indistinguishable in terms of their effect size and confidence intervals (33). Moreover, disorganization has also been linked to caregivers' withdrawal and dissociative behaviors (39, 40) or hostile–helpless states of mind, possibly due to the caregiver's own traumatic experiences (41, 42). A further case in point is the context of institutionalization or prolonged caregiver separation where the need for a continuously available and reliable caregiver is experienced over a long period without any hope of being met (“activation without assuagement”), resulting in resignation and despair (43, 44). Especially in early childhood, caregivers are the main source of co-regulation of mild to overwhelming affective states (safe haven function). Hence, prolonged absence of or chronically rebuffing caregivers, as well as other major unpredictable discontinuities in the caregiving context (e.g., multiple changing caregivers), bears the potential to disrupt normative development of organized attachment. This dovetails with meta-analytic data showing that over half of institutionalized children are classified as disorganized (31, 45).

Surveying different populations, while disorganization occasionally occurs within middle-class samples (infants:15%, adults: 18%; “unresolved–disorganized state of mind”), prevalence estimates are higher among samples burdened by sociodemographic risks (e.g., offspring of teen mothers: 23%, families with low socioeconomic status: 25%) and yet higher still among samples with clinical or psychosocial risks (clinical adult samples: 43%, children with neurological abnormalities: 35%, adoptees: 31%, offspring of caregivers with substance abuse: 43%, previously institutionalized samples: 54–73%, and children raised by maltreating caregivers: 48–90%) (31, 34, 45–47)^2^. Despite these elevated rates of disorganization in samples exposed to adversity, the mapping of adversity with disorganization is far from perfect, suggesting that disorganization may account for meaningful variance over and above adversity. Thus, for example, in women with a history of childhood abuse, attachment disorganization gave rise to a 7½-fold increase in the odds of being diagnosed with post-traumatic stress disorder (48), stressing its putative role in the aftermath of adversity, where disorganization is thought to act akin to an intermediary factor, signaling how adaptively trauma has been processed (4, 49, 50).

Conceptualizing attachment disorganization as a potential intermediary process may also help explain a salient pattern emerging from recent research—including large-scale studies and meta-analyses (51–55)—documenting unique and especially toxic effects for mental health following emotional maltreatment, in particular [e.g., persistent rejection or absence of support from the caregiver; see (56)]. Mounting evidence thus suggests that the pathogenic effects of emotional maltreatment (e.g., on depression) may exceed and potentially even explain those of other (physical) subtypes of maltreatment. To account for this pattern, many scholars invoke conceptual links between emotional maltreatment and attachment disorganization as well as impaired reflective functioning (54, 55, 57, 58). Supporting these ideas, the maltreatment-related risk for attachment disorganization is mitigated when abuse and neglect transpire in the context of emotionally supportive caregiving relationships (58, 59). In keeping with this, scholars contend that a “pathogenic relational experience” may often lie at the core of child maltreatment (60, 61), potentially reflecting a seedbed for other forms of maltreatment to occur.

### Hyper- and Hypo-Arousal Pathways to Disorganization

The brief summary presented above bolsters the view of disorganization as a heterogeneous phenomenon. Thus, many divergent behaviors (e.g., contradictory, freezing, apprehensive behaviors in the presence of caregivers) and narrative indicators (e.g., sudden affective shifts, incompatible affect, interrupted speech, bizarre descriptions, lapses in reasoning when recounting loss or trauma) pertain to the classification of individuals as disorganized in childhood and adulthood (4, 62, 63). Specifically, in the case of narratives, organized strategies for coherently discussing trauma suddenly collapse as the memory of the traumatic experience is thought to become frighteningly imminent and overwhelming (fright without solution), impeding ongoing mental processes (64). Moreover, multiple distinct forms of and pathways to disorganization have been proposed in the literature (34, 38, 65) and may even have been anticipated in early unpublished writings of Bowlby (43).

**Figure 1:**
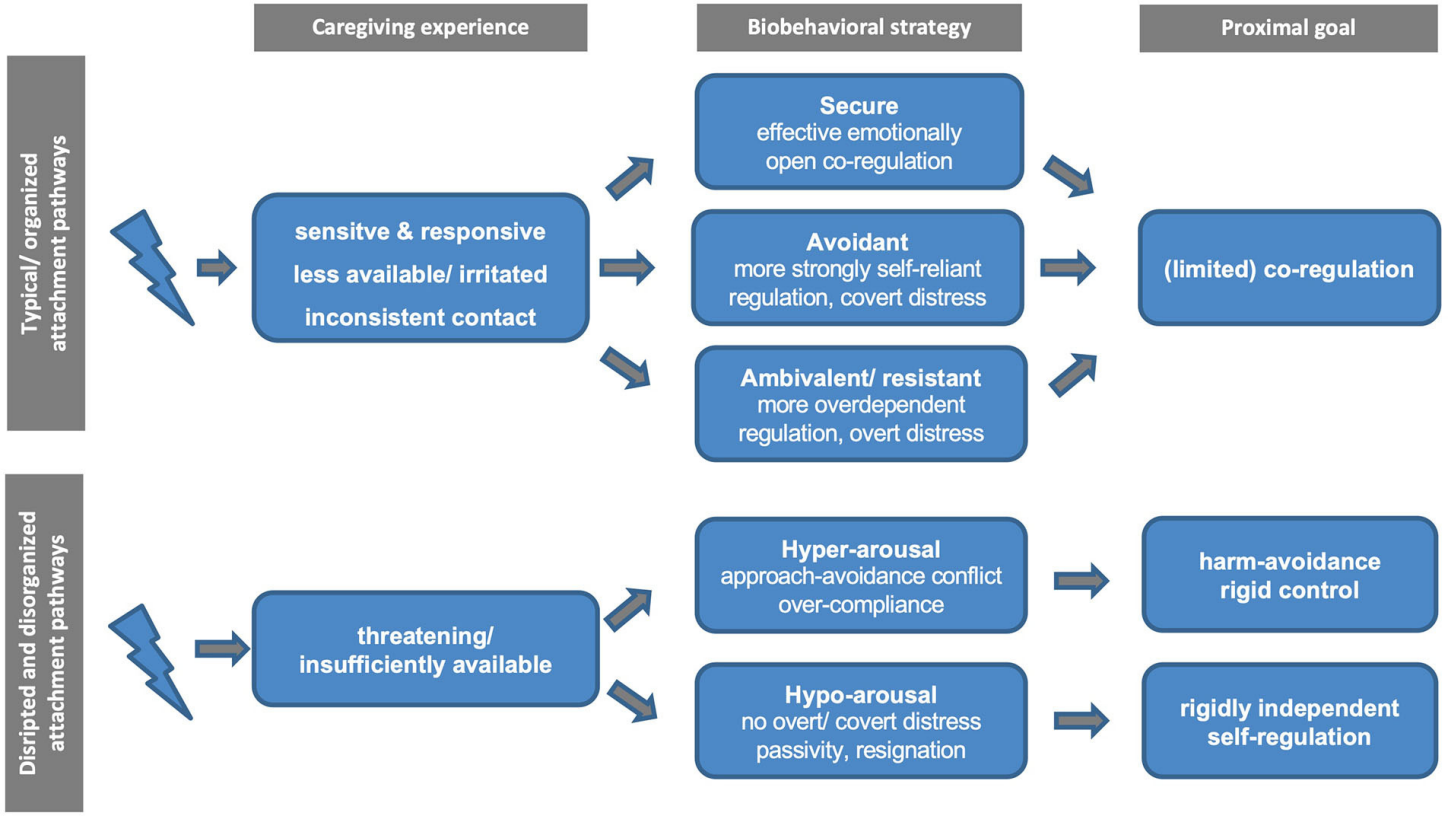
Schematic diagram of the proposed typical vs. disrupted and disorganized attachment pathways.

Attempting to come to terms with this heterogeneity, shortly after the notion of disorganization was first introduced, Crittenden and Ainsworth (66)^3^ highlighted the added value of distinguishing between abuse and neglect in the context of discussing attachment disorganization. For example, the abused child is “locked into forming an attachment to his primary caregiver and yet his experience teaches him that this attachment figure may be a source of pain and injury” [(66), p. 449]. Conversely, neglected children “desperately need the comfort and support of others [but] rarely seek it or seem comforted by it when they receive it” [(66), p. 450]. In line with these proposals and recent efforts to delineate different pathways to disorganization, Figure 1 outlines two distinct neurobiological hyper- and hypo-arousal phenotypes in the context of disrupted and disorganized attachment. Importantly, while these pathways are informed by current neural models of adversity, threat, and deprivation (67–70), they remain to be further examined and empirically substantiated, particularly in the case of disrupted and disorganized attachment. Accordingly, the proximate attachment-oriented mechanism of co-regulation by caregivers is thought to be severely impaired for both hyper- and hypo-arousal pathways and subordinated to harm avoidance and rigid self-regulation, respectively. Nevertheless, we believe that these behaviors serve as the best possible solution for promoting survival in the context of insufficiently available or threatening primary caregivers (who exhibit frightened/frightening behaviors).

### Summary

As a point of departure, we provided a brief overview of disorganized attachment, beginning with key theories and evidence regarding its putative origins and sequelae before turning to its inherent heterogeneity. The heterogeneity of disorganization emerges not only in terms of its phenomenology but also regarding its ontogeny and etiology and may at least partly reflect distinct adaptations upon exposure to abusing and/or neglecting caregivers. Analogous to early and current work on attachment disorganization and recent developments in neuroscience (see below), we consequently propose a distinction between a hypo- and hyper-arousal subtype primarily deriving from the caregiver serving either as a threatening or as an insufficient source of co-regulation, respectively.

In the next section, before elaborating on the possible neurobiological underpinnings of disrupted and disorganized attachment, we offer a brief summary of NAMA's functional neuro-anatomical account of organized human attachment within the field of social neuroscience. Readers familiar with the up-to-date version of NAMA (17) are referred directly to the section on “The Social Neuroscience of Disrupted and Disorganized Attachment.”

## The Social Neuroscience of Organized Human Attachment

**Figure 2:**
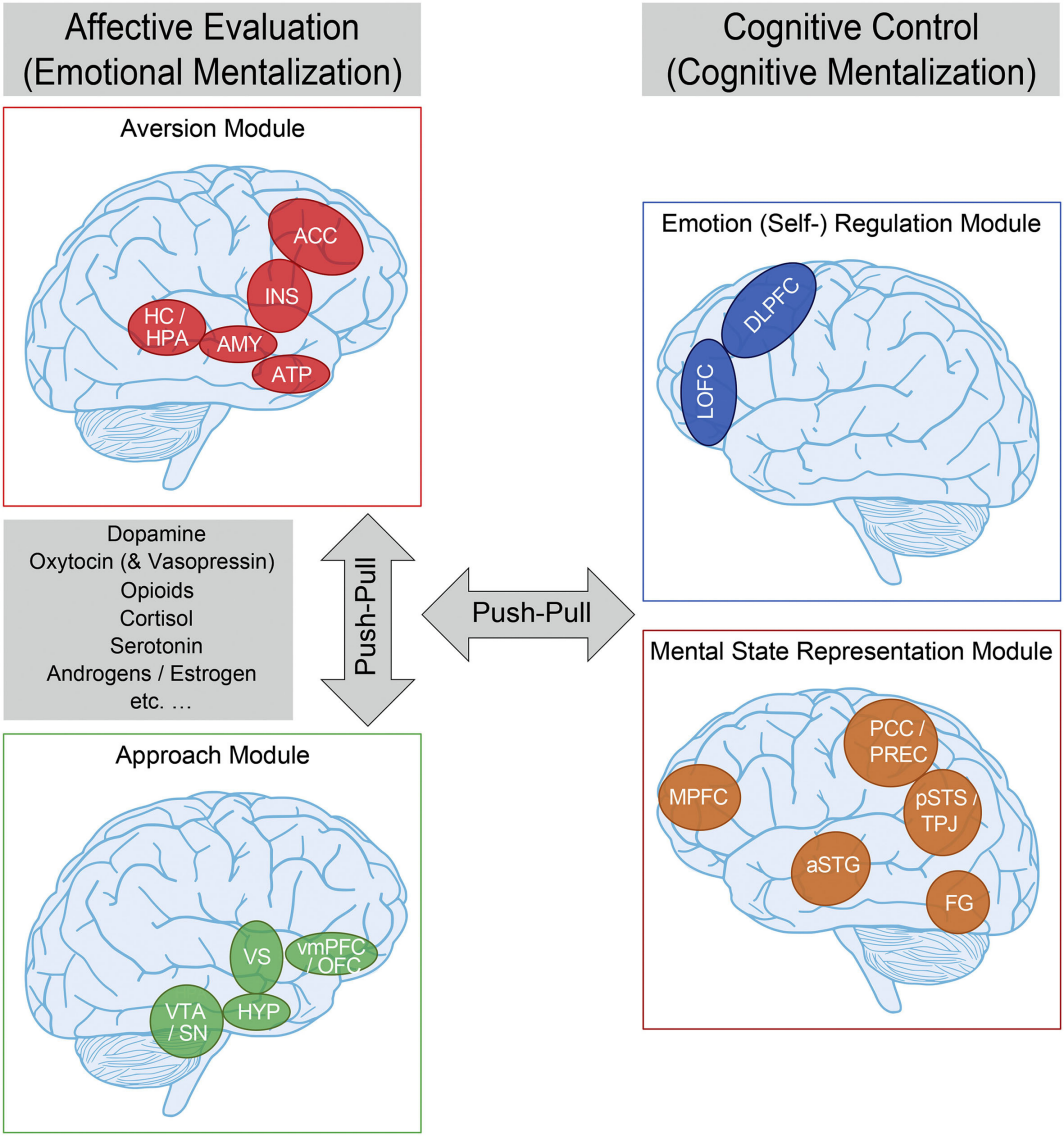
Functional neuro-anatomical model of human (organized) attachment (NAMA). We propose that the (organized) human attachment system can be described by two affective/emotional (left) vs. cognitive/control (right) systems on the neural level, and that these systems can be further separated into two modules each (affective evaluation: aversion—red—and approach—green; cognitive control: emotion regulation—blue—and mental state representation—orange). We further suggest that the aversion and approach modules as part of the affective system, as well as the affective and cognitive systems are in a dynamic “push-pull” balance. Finally, we propose that neural activity within the affective system is mediated by (amongst others) dopamine, oxytocin (and vasopressin), endogenous opioids, cortisol, serotonin, androgens/estrogen, etc. aversion module—ACC, anterior cingulate cortex; INS, insula; HC/HPA, hippocampus/HPA-axis; AMY, amygdala; ATP, anterior temporal pole; approach module—vmPFC/OFC, ventromedial prefrontal/orbitofrontal cortex; HYP, hypothalamus; VTA/SN, ventral tegmental area/substantia nigra; emotion regulation module—DLPFC, dorsolateral prefrontal cortex; LOFC, lateral orbitofrontal cortex; mental state representation module—MPFC, medial prefrontal cortex; PCC/PREC, posterior cingulate cortex/precuneus; pSTS/TPJ, posterior superior temporal sulcus/temporo-parietal junction; aSTG, anterior superior temporal gyrus; FG, fusiform gyrus. For more information, please refer to the main text. Adapted from Long et al. (17) and based on Vrtička (15) and Vrtička and Vuilleumier (16).

Most theoretical accounts of the neurobiological substrates of interpersonal interactions and relationships derived from social neuroscience thus far only indirectly refer to attachment theory. This likely reflects the fact that only a limited number of social neuroscience studies assess attachment using narrative or self-report measures (71), and extant work has nearly exclusively focused on adult populations. Nevertheless, we recently synthesized all available experimental evidence, suggesting a comprehensive framework of the social neuroscience of (organized) human attachment (functional neuro-anatomical model of human attachment—NAMA; Figure 2) (15–17). NAMA draws directly on attachment theory in that it presupposes a prototypical attachment pathway with several sequential components that constitute the proposed underlying neurobiological and brain mechanisms of organized (i.e., secure, avoidant, anxious–ambivalent) human attachment.

### Prototypical Attachment Pathways and Neuro-Anatomical Model

In keeping with attachment theory (19–22), we assume that (external or internal) events appraised as threatening reliably activate the attachment behavioral system. Such threat appraisal—and associated appropriate fear response—is thought to challenge homeostasis, necessitating a compensatory physiological and behavioral response to (re-)gain an optimal internal milieu. Following the notion of allostasis (72), this regulatory process helps the organism to adapt to changes in the environment and meet anticipated demands. Accordingly, we postulate the presence of an *aversion module* in NAMA that encodes negative social experiences—from social exclusion or abandonment in times of need to any kind of negative occurrences, including those of a non-social nature—in terms of a neural relevance/salience signal (73), prompting further action (i.e., allostatic regulation). At the level of neurotransmitters/hormones, the primary stress-related hormone cortisol, acting through the hypothalamic–pituitary–adrenocortical (HPA) axis, may underpin such aversion module activation (Figure 2).

Once the fear response has been triggered, the next crucial element of a prototypical attachment pathway involves proximity seeking maintained by a fundamental social approach motivation. In other words, we propose a “social flight response” (74), not unlike the tend-and-befriend responses postulated elsewhere (75), but tailored more specifically to AFs. The underlying notion of this approach motivation is that (mutual) social interactions should be subjectively experienced and neurally encoded as intrinsically rewarding. We therefore situate a reward-related *approach module* associated with the action of, among others, dopamine, oxytocin, and endogenous opioids as the second of four modules in NAMA (Figure 2).

Both the approach and aversion modules are deemed to be activated by, and represent more automatic, bottom-up biological and neural mechanisms and are thus summarized as *affective evaluation* or *emotional mentalization processes* (76). It should also be noted here that we view the approach and aversion modules as two rather independent—albeit complementary—neurobiological systems that can be de- or hyper-activated to varying degrees in attachment-relevant situations as a function of inter-individual differences in secure vs. insecure attachment orientations (even in opposing directions), that is, we do not equate de- or hyper-activation of the approach module with attachment security and de- or hyper-activation of the aversion module with insecurity as two diametrically opposing ends of one single attachment dimension. Furthermore, we believe that, except during the initial moment of approach module involvement, to motivate a social approach response of support seeking under distress (i.e., during simultaneous aversion module activation), for typical (or organized) attachment patterns, the two emotional modules should not be activated concomitantly for an extensive time period/chronically, as this would lead to conflicting social emotional states.

Once social proximity has been successfully established (and the source of threat has been abolished), NAMA suggests that the next stage in the prototypical attachment pathway can unfold: *emotion regulation*. Initially mainly accomplished by external co-regulation through AFs, this is increasingly supplanted by self-regulation (i.e., by virtue of an internalized source of regulation) with advancing development, with both decelerated and accelerated adoption of self-regulation associated with suboptimal outcomes (69). The primary goal of the emotion regulation module is to down-regulate negative emotional states to re-establish homeostasis and thereby reduce the allostatic load. In the context of attachment, it has been elegantly demonstrated that such regulatory influence of emotion regulation (mainly *via* the aversion module) can encompass both conscious and unconscious mechanisms and relies upon a variety of emotion regulation strategies (77–79).

Provided that emotion regulation is effective and a return to homeostasis is achieved, re-activation of the approach module may occur following NAMA. This is because we assume that the return to the organism's optimal inner milieu and normal range of arousal (entailing a reduced allostatic load) through effective affect co- or self-regulation is experienced as positive *per se*. Such personal positive experience of physically calming down is presumably accompanied by additional socially positive aspects of the interaction with the external co-regulator [e.g., affective touch, soothing verbalizations, etc. (72)] that serve to establish a feeling of safety and security, which further reinforces the rewarding nature particularly of co-regulation and the social interaction as a whole.

Finally, we posit a *mental state representation* module in NAMA. In the context of attachment, the mental state representation module is conceived of as a central part of the neural substrate of internal attachment working models that emerge through repeated interactions with others and comprise predictions about how to approach whom in times of need, how the approached individual(s) will respond, and whether their reaction will be helpful or not. Social neuroscience postulates that a so-called default mode network may maintain such processes [(80), see Figure 2].

Both the emotion regulation and mental state representation modules are summarized as *cognitive control* or *cognitive mentalization* processes in NAMA [see (76)]. They are thought to modulate the perception of social emotional cues and thus emotional mentalization processes through top-down influences by down- and up-regulating emotional states and determining social approach or aversion motivations. Within this context, we refer to mentalization as the imaginative mental activity that enables us to perceive and interpret human behavior in terms of intentional mental states [e.g., needs, desires, feelings, beliefs, and goals; see (51)]. Broadly speaking, it is thought that emotional and cognitive mentalization processes are in a dynamic balance and that the “switch point” between them is determined by the magnitude of affective arousal related to attachment system activation in association with the respective individual attachment-related strategies to maintain successful regulation. Consequently, high affective arousal should push the “switch point” toward emotional mentalization and thus more rigid, fast, and unconscious processing [(76); for neural and behavioral evidence in adults and children see (81, 82), respectively; see Figure 2].

### Inter-Individual Differences in Organized Attachment

Besides describing the fundamental biological and neural building blocks of human attachment associated with a prototypical attachment pathway (Figure 2), we place particular emphasis on how inter-individual differences in the three organized secure vs. insecure—avoidant and anxious–ambivalent—attachment orientations affect the functioning of the four NAMA modules in healthy participants across the lifespan. In so doing, several patterns appear to emerge, which are briefly summarized below and in Figure 2 [for more details and a comprehensive summary of the evidence base, please see (15–17)].

Firstly, *secure attachment* appears to involve reduced aversion module activation during stressful situations (especially when under threat or in pain) and preserved aversion module structural integrity (comprising the HPA stress axis) in the long term. Both mechanisms are likely propagated *via* a protective effect of initially readily available social resources for co-regulation, eventually translating into more efficient self-regulation (by means of an internalized source of regulation), and enhanced by security priming. This explanation is bolstered by positive representations of others in the approach module and more extensive functional connectivity between the emotional and cognitive mentalization modules of NAMA sustaining self-regulation and mental accessibility of others.

Secondly, *attachment avoidance* and its associated de-activating strategies appear to be most consistently linked to altered approach module functionality because (mutual) social interactions with others are subjectively (i.e., pleasantness ratings), biologically (i.e., oxytocin and opioid signaling), and neurally encoded as less rewarding. Additionally, although aversion module activation during negative social information processing is reduced under specific circumstances (particularly during brief and mild social exclusion in children and adults—likely due to negative expectancy and ensuing disengagement) (83–85), it is typically increased due to inefficient self-regulation (mainly through suppression) (86) and lower availability of social resources to deal with distress (e.g., lengthy social exclusion, especially in adolescence) (87). The latter also manifests by altered aversion module structure and connectivity, epigenetic modification of the HPA stress axis, accelerated biological aging/reduced telomere length, and increased baseline bodily readiness (i.e., higher fasting glucose levels) (88, 89), all indicative of heightened self-reliance and associated chronic stress. The widespread general association between attachment avoidance and the presence of de-activating secondary strategies therefore appears to only partially “succeed” at a neurobiological level.

Finally, *anxious–ambivalent attachment* characterized by hyper-activating strategies also associates with increased aversion module activation during negative social information processing and altered aversion module structure and connectivity. There are, however, no consistent indications of a systematic regulation inefficiency and/or chronic stress on the epigenetic level (HPA stress axis). This pattern related to attachment anxiety therefore rather points to increased saliency processing of social cues, indicating the unavailability of others and a dependence on external (co-)regulation. Such notions are corroborated by increased approach module activation to (unexpected) positive social clues reflecting a sustained wish for social closeness and care when in need.

It should be mentioned here that, in contrast to data on the aversion, approach, and emotion regulation modules, findings implicating the mental state representation module linked to attachment avoidance and anxiety are still too sparse for deriving solid conclusions. We are only aware of one study in adults linking avoidance with neural correlates, reflecting hypo-mentalization during a specific mentalization task, and one study in adolescents associating anxiety with decreases and increases in brain activity during self- and other-representation in a range of areas [also outside the mental state representation module; see (17) for details].

### From First- to Second-Person Social Neuroscience of Attachment

Most of the aforementioned patterns of findings draw on data gathered by only obtaining behavioral, biological, and brain measures from one participant (i.e., first-person social neuroscience). During the previous years, however, there has been a paradigm shift toward assessing such measures from two (or more) directly interacting participants (i.e., second-person social neuroscience). In so doing, a special focus is directed toward bio-behavioral synchrony—the time-locked attunement of behavioral, physiological, endocrine, and neural responding—during or immediately after social interaction (90). One prominent social neuroscience method to assess neural attunement in terms of inter-brain coherence is functional near-infrared spectroscopy (fNIRS). In line with the theoretical assumption put forward by Feldman (90), a stronger increase in inter-brain coherence during cooperative tasks is usually found between close interaction partners such as mother–child dyads or romantic couples [as compared to interactions between strangers (91, 92)]. Such results, however, do not allow for directly answering the question whether and, if yes, how, inter-individual differences in relationship quality (i.e., attachment) may influence bio-behavioral synchrony/inter-brain coherence during cooperative tasks within a given interaction partner category. To our knowledge, there are only two fNIRS studies available to date that provide preliminary evidence toward this end.

In a first study, inter-brain coherence during a cooperative button press task within mother–child dyads (child age 8–12 years) was found to be reduced among children with an avoidant attachment toward their mothers (93). These findings, however, did not survive correction for multiple comparison and child gender, age, and attachment anxiety scores. In a second study, inter-brain coherence was assessed during an interactive problem solving task (tangram puzzle) in mothers with their 5 year-old children (94). Besides finding that inter-brain coherence during cooperation was positively associated with task performance, it also correlated positively with behavioral measures reflecting a secure mother–child relationship, such as behavioral reciprocity and child agency. Taken together, these data suggest that a more secure relationship can also manifest itself by increased bio-behavioral synchrony during direct interaction. More research, however, is needed to further extend and replicate these preliminary findings in an attachment context.

### Summary

Within NAMA, we propose a prototypical initial attachment pathway and its translation into four fundamental biological and neural building blocks of human attachment—the four aversion, approach, emotion regulation, and mental state representation modules. This framework provides the foundation for the three organized secure, avoidant, and anxious attachment pathway derivatives and how the associated inter-individual differences affect the functioning of the four NAMA modules in healthy participants across the lifespan. As more recent investigations try to establish links between bio-behavioral synchrony and inter-individual differences in attachment in two (or more) interacting individuals, the social neuroscience of attachment is currently entering a new era.

In contrast to the aforementioned emerging patterns relating to organized secure, avoidant, and anxious–ambivalent attachment, much less is known about the social neuroscience of maltreatment-related disruption and disorganization of attachment. One central question is whether attachment disorganization and/or maltreatment may manifest comparably to attachment avoidance and/or anxiety on a biological and brain level. Ideally, the evidence already available from healthy participants summarized in NAMA may serve as a point of reference for interpreting the data thus far available using social neuroscience paradigms in clinical populations and generating future investigations to further characterize the biological and neural signatures of maltreatment-related disruption and disorganization of attachment.

## The Social Neuroscience of Disrupted and Disorganized Attachment

**Figure 3:**
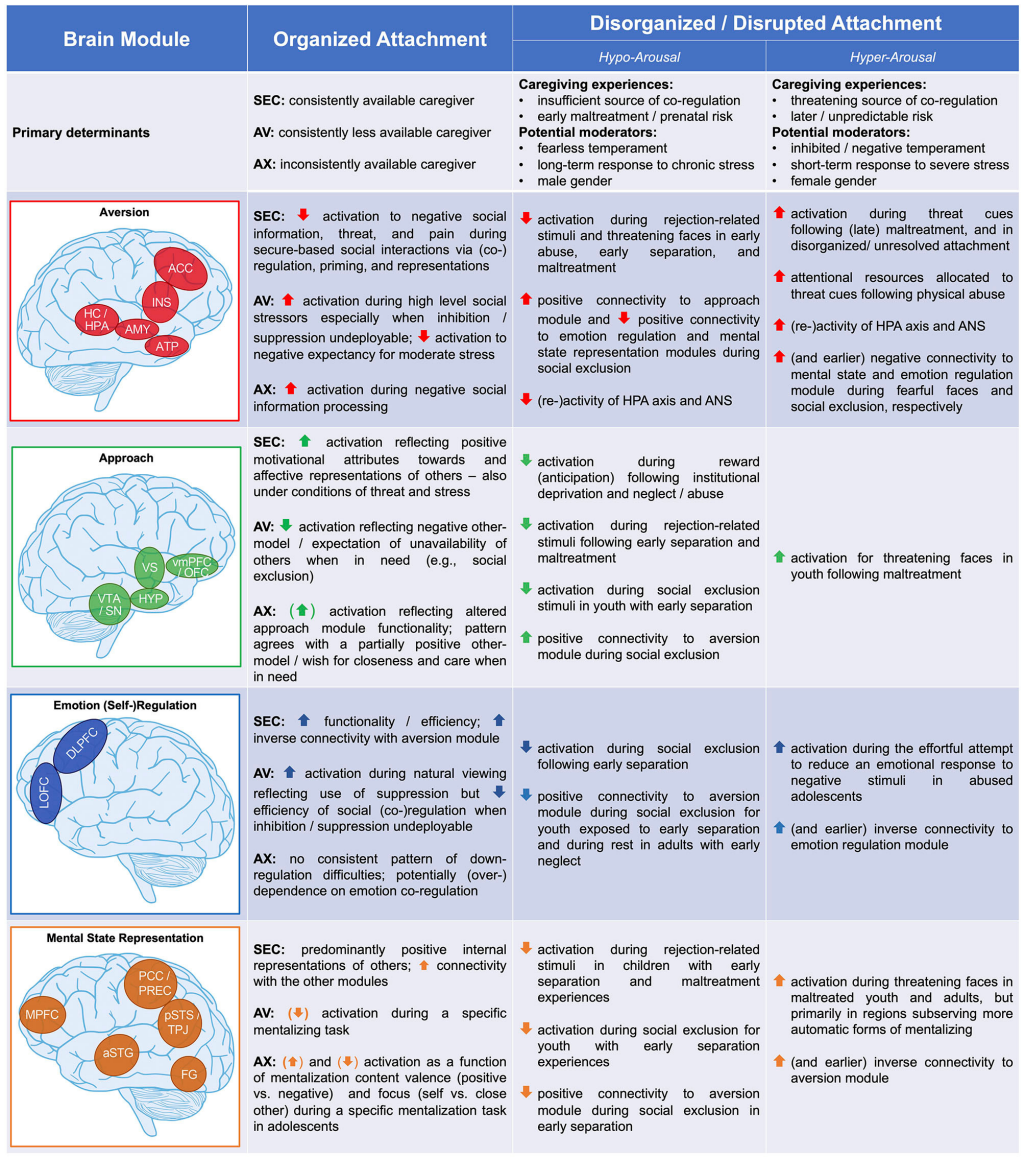
Functional neuro-anatomical model of disrupted attachment (NAMDA). By integrating theoretical models and empirical evidence from the fields of attachment and childhood maltreatment, we propose that disruption and disorganization of attachment manifest in two differential neurobiological phenotypes characterized by hypo-arousal vs. hyper-arousal. Empirical support for these neurobiological phenotypes is summarized focusing on brain function of four neural modules—the aversion, approach, emotion regulation, and mental state representation modules—and compared to the neurobiological underpinnings of organized secure, avoidant, and anxious-ambivalent attachment as formulated in the functional neuro-anatomical model of human (organized) attachment (NAMA). Further, primary determinants of organized and disorganized attachment are listed. aversion module—ACC, anterior cingulate cortex; INS, insula; HC/HPA, hippocampus/hypothalamic-pituitary-adrenal axis; AMY, amygdala; ATP, anterior temporal pole; approach module—vmPFC/OFC, ventromedial prefrontal/orbitofrontal cortex; HYP, hypothalamus; VTA/SN, ventral tegmental area/substantia nigra; emotion regulation module—DLPFC, dorsolateral prefrontal cortex; LOFC, lateral orbitofrontal cortex; mental state representation module—MPFC, medial prefrontal cortex; PCC/PREC, posterior cingulate cortex/precuneus; pSTS/TPJ, posterior superior temporal sulcus/temporo-parietal junction; aSTG, anterior superior temporal gyrus; FG, fusiform gyrus; ANS, autonomic nervous system; AV, avoidant attachment; AX, anxious-ambivalent attachment; SEC, secure attachment. Adapted from Long et al. (17).

As outlined above, our aim is to extend NAMA—the model of organized attachment outlined in the previous section—to disrupted and disorganized attachment in the context of maltreatment and adverse attachment-related experiences. To this end, we draw on models of structural and functional brain alterations in the wake of early adversity (67–70). Informed by some of these models (67, 68), we propose distinguishing between a neurobiological *hyper-arousal* phenotype related to primary caregiver(s) as a source(s) of threat (e.g., abuse) and a neurobiological *hypo-arousal* phenotype as a consequence of (early) distress unassuaged by caregiver(s) (e.g., emotional neglect). In so doing, we feel that it is particularly pertinent to point out that we are by no means equating these phenotypes with concrete adverse events, specifically abuse (the presence of threatening/harmful input) and neglect (or deprivation/lack of necessary input), respectively (95). In our view, the fundamental issue rather is if these adversities are mainly attributable to actions by the primary caregivers and the attendant issue of whether the adversities interfere with the function of caregivers as sources of co-regulation. As such, pervasive abuse and neglect may serve as prototypical environmental experiences that often coincide with the expression of these neurobiological phenotypes, yet other dimensions such as timing of adversity [e.g., (96)], child gender (97), neonatal hippocampal volume (11), temperament, or genotype (4, 23) may prove as crucial moderators (see Figure 3, row 1).

Thus, as already elegantly outlined by Crittenden and Ainsworth (66), unlike exposure to abuse and neglect, disorganized attachment is conceptualized in terms of a representational model amalgamated from the history of caregiving experiences (i.e., not a singular or set of singular event/s) as well as the individual's adaptive and (co-)regulatory efforts marshaled in response to these experiences. This is not to deny that adversity cannot have a lawful and direct temporary or lasting impact on neurobiological development as a function of the specific patterning of experience regarding, for example, the timing of experience in terms of sensitive periods of brain development (70). However, the recent data from the Bucharest Early Intervention Project (98) and English and Romanian Adoptees study (99) provide first causal evidence in humans that sensitive periods and windows of opportunity regarding the development of the social brain appear to be broader relative to those of other species [see also (100)]. Thus, the impact of severe and chronic deprivation seems at least partly reversible if it is terminated early (101), and puberty may provide yet another window of opportunity for potential recalibration (102). In turn, this suggests that developmental time windows exist, during which effects of even such severe adversities remain highly malleable and under the influence of subsequent caregiving experience.

Informed by the ecophenotype model of Teicher et al. (70, 103), we assert that this perspective on the neural correlates of early adversity may offer a helpful new vantage point, potentially aiding us in understanding the many (initially) adaptive behaviors children and adults show in the face of adversity, including hyper-cooperativeness (104), compulsive compliance (105), and indiscriminate friendliness (106), which would otherwise remain puzzling from a pure perspective of neuro-cognitive dysfunction [see (107) for evolutionary arguments on why these behaviors might be adaptive, for example, in the sense of minimizing the odds of malignant and maximizing the odds of benign interactions]. In particular, we propose distinguishing between neurobiological hyper- and hypo-arousal phenotypes coinciding with disrupted and/or disorganized attachment, primarily based on the available neurobiological data from children with severe adversity. Importantly, these admittedly speculative and preliminary assertions are largely based on *indirect* evidence from samples exposed to severe early-life adversity rather than *direct* evidence from effects of attachment disorganization, a distinction that we will repeatedly return to below (and that is summarized in Figure 3, rows 2–5).

### Alterations in the Aversion Module

Most neurobiological alterations *directly* associated with disorganized attachment have been documented in neural regions and physiological indices linked to what has been termed the aversion module in NAMA. For example, a number of psychophysiological studies suggest that infants classified as disorganized show increased reactivity of the autonomic nervous system and HPA axis to caregiver separation and reunion procedures relative to infants classified in one of the organized categories (108–110).

Importantly, these findings, indicative of a hyper-reactive HPA axis, dovetail with the pattern observed in children and adolescents in the wake of severe physical or sexual abuse but not neglect (111, 112). However, by far the largest population-based Generation-R study comparing cortisol responses to the Strange Situation Procedure (SSP) among 72 disorganized to 297 non-disorganized infants failed to confirm this pattern, rather showing that anxious–ambivalent infants exhibited the highest cortisol reactivity relative to other classifications [(113), see (114) for a second non-replication]. That said, although the Generation-R Study is one of the largest of its kind, presumably due to its population-based nature, there was a high proportion of disorganized infants who received a secondary secure classification, potentially suggesting that their disorganized status was less attributable to severe abuse or neglect [see (4, 115, 116)], though this also applied to studies which detected cortisol hyper-responsivity among disorganized infants (110). Moreover, it is noteworthy that Generation-R employed an adapted SSP with shorter (pre)separation episodes, which may have diminished the odds of detecting evidence of HPA axis hyper-reactivity.

Two functional magnetic resonance imaging (fMRI) studies showing distressing attachment-related picture stimuli to unresolved–disorganized adults demonstrated increased amygdala activation compared to their organized counterparts (13, 14). Interestingly, the latter resembles the pattern of increased amygdala activation in response to threatening faces among abused vs. non-abused youth (117), with a recent meta-analysis indicating that this pattern applies across maltreated children and adults alike (118). These data directly implicate the heightened activity and responsivity of threat detection and stress response during activation of the attachment system related to disorganized attachment, which could partly account for the persistent freezing and/or apprehension of these infants in response to their caregivers.

Moreover, in a small study of 18 infants from low-income families followed through adulthood, Lyons-Ruth et al. (9) found that disorganized attachment classified using the SSP in infancy was associated with a larger amygdala volume in adulthood, while a recent study on adults (*N* = 74) showed unresolved attachment to be associated with reduced hippocampal volume (12). Similar morphological changes have again surfaced in human and non-human primate studies showing increased amygdala volume following exposure to physical abuse (119), chronic maternal depression (120), as well as institutional rearing and international adoption (121, 122).

These patterns notwithstanding, there is also some direct support for opposing effects of disorganization, indicating the presence of a hypo-arousal phenotype. Firstly, disorganization coincided with a flattened diurnal cortisol slope in infancy in the aforementioned Generation-R study, with follow-up analyses implicating a hypocortisolism that applied particularly to the disorganized children with an insecure rather than a secure secondary classification (113). Crucially, at the level of the HPA axis, large-scale studies have recently documented that maltreatment, in particular when occurring early and involving neglect by the caregiver, is linked with hypocortisolism (123–126). This tamping down or “blunting” of indices composing the aversion module may reflect the long-term consequences of an “evolutionarily conservative” response involving an excessively self-reliant emotion regulation strategy that is metabolically less costly and minimizes the risk inherent in depending on others as sources of co-regulation (127, 128).

Secondly, the largest recent structural neuroimaging study in over 500 children with infant attachment indexed by the SSP found that disorganized attachment at 14 months was directly linked to 10 year-olds' increased hippocampal volume as well as tentative indications of increased structural integrity of the uncinate fasciculus—the largest white matter tract connecting the prefrontal cortex and the anterior temporal lobe (though the latter finding did not survive correction for multiple testing) (8). Intriguingly, the latter may resemble a stress-dependent acceleration of neural development and prefrontal–amygdala connectivity, in particular, as documented in previously institutionalized youth (69). Potentially in a similar vein, enhanced functional connectivity between anterior medial temporal gyrus and amygdala has also been associated with adverse childhood experiences, with physical and emotional neglect constituting the most important subtypes (129). Moreover, indirect evidence stems from fMRI studies administering rejection–stimuli and a social exclusion task to youth who primarily experienced emotional abuse and neglect, documenting diminished activation of the amygdala and dorsal anterior cingulate (130, 131).

This latter pattern of hypo-arousal may prove particularly distinct compared to the organized insecure attachment classifications outlined in NAMA. Here we also posit a divergent pattern *vis*-à-*vis* insecure–avoidant individuals whose suppressing strategies primarily are less efficient during excessive, persistent, or inescapable threat (e.g., during the SSP itself or lengthy social exclusion in adolescence). Unlike for avoidance, we predict that the disorganized hypo-arousal phenotype may exhibit reduced aversion/stress responses even during such high-level stressors, such as the Trier Social Stress Test, where early deprivation has been associated with a blunted cortisol response (132). By contrast, we hypothesize that activation in physiological and neural markers of aversion and distress characteristic of the hyper-arousal phenotype will be more pronounced than in the organized attachment classifications as a whole, though the effects are likely to prove least strong *vis*-à-*vis* organized insecure-ambivalent (i.e., anxious) strategies.

### Alterations in the Approach Module

To the best of our knowledge, little or no *direct* evidence exists to date for effects of disorganization on brain regions comprising the approach module in NAMA. However, results from the large-scale Generation-R sample of 626 6 week-old infants of whom 132 were later classified as disorganized (vs. organized) at 14 months in the SSP revealed reduced gangliothalamic ovoid diameter, which may potentially also reflect structural alterations in (early) basal ganglia development (133). Similarly, ample *indirect* evidence suggests the diminished responsiveness of the basal ganglia (mainly ventral striatum) in response to (anticipation of) reward, primarily among youth exposed to severe deprivation or neglect (134, 135) as well as family adversity (136). Though one study also linked childhood abuse to reduction of globus pallidus activation during reward anticipation, the probable concomitant effects of neglect were not assessed in this study (137). Broadly speaking, this blunted approach-related response may reflect a motivational deficit impeding effective engagement with environmental pressures (138) which, we suggest, may also reflect reduced gravitation to sources of co-regulation in childhood. Coupled with the aforementioned blunting of systems involved in the aversion module, diminished reward sensitivity and approach reactivity may account for Crittenden and Ainsworth's (66) prescient observation that neglected children fail to act on the need for co-regulation from their caregivers.

Besides this, it is intriguing that a meta-analysis on maltreated youth and adults specifically suggested increased basal ganglia activation (globus pallidus and lentiform nucleus) during exposure to threatening faces (118). Furthermore, in a sample of children with early caregiver separation experiences (over half of this sample exposed to neglect prior to separation), Puetz and colleagues (130) documented a greater activation of the ventral tegmental area (VTA) and increased functional connectivity of VTA to dACC among youth during social exclusion, though the caudate nucleus showed reduced activation.

Tentatively, we interpret the activation of regions in the approach module during exposure to aversive stimuli as neural evidence of an approach–avoidance conflict, in particular, when it occurs in conjunction with activation of regions linked to aversion [as suggested by VTA–dACC connectivity in (130)]. It will be incumbent on future research to determine whether such patterns are also observable at other physiological levels, such as the potential for co-activation of parasympathetic and sympathetic branches of the autonomic nervous system implicit in the notion of autonomic space proposed by Berntson et al. (139). To the extent that Cyr et al. (34) link the approach–avoidance conflict more specifically with abuse, due to the dual role of the caregiver as safe haven and source of distress, it is conceivable that such patterns will prove more characteristic of the hyper-arousal phenotype of disrupted and disorganized attachment. That said, we noted at the outset that absence of and persistent rebuffs by the caregiver may also coincide with such conflict because the need for the caregiver becomes associated with alarm (even if s/he is not necessarily the source)—which may suggest that an approach–avoidance conflict characterizes both hypo- and hyper-arousal phenotypes.

### Alterations in Emotion Regulation and Mental State Representation Modules

Given the paucity of *direct* and *indirect* evidence regarding the effects of attachment disruption on brain regions associated with emotion (self-)regulation and mental state representation modules as well as their structural and functional overlap, we will discuss these jointly. Regarding emotion (self-)regulation, the aforementioned study on youth with early separation experiences showed a diminished activation of the dorsolateral prefrontal cortex (DLPFC) during social exclusion (130). Furthermore, the same and a related study exposing maltreated youth to rejection-related verbal stimuli detected a diminished activation in regions linked to mental state representation [medial PFC (mPFC), temporo-parietal junction, and precuneus], though findings on superior temporal sulcus (STS) were contradictory, with activation increased in one (130) but decreased in another study (131), potentially due to task- or sample-specific factors. Most children in the latter study (131) experienced emotional abuse, followed by neglect and witnessing domestic violence, whereas most children in the former study (130) had been separated from their caregivers, which is usually an indication of severe multiple-subtype maltreatment, but it was only reported that 64% of their sample had experienced some form of neglect. Broadly speaking, we would therefore tentatively link the hypo-arousal phenotype to diminished activation in regions subserving mental state representation, especially during social stress, potentially analogous to the mentalizing deficits often linked with attachment disorganization and related disorders (140, 141).

By contrast, McLaughlin et al. detected increased DLPFC, mPFC, and dACC activation among abused adolescents during the effortful attempt to reduce an emotional response to negative stimuli, potentially indicating a less efficient emotion regulation region, as indicative of the hyper-arousal phenotype (142). Furthermore, the aforementioned meta-analysis by Hein and Monk (118) found an increased activation in posterior STS (pSTS) during exposure to threatening faces among maltreated youth and adults relative to non-maltreated controls. It is noteworthy that while the pSTS is thought to perform a central role across most, if not all, forms of social perception, meta-analytic data suggest an intermediate-level role between automatic/reflexive and effortful/controlled mentalizing, aiding, for example, in the inference of intentions from behavior (143). Notably, this contrasts markedly with the more controlled/effortful forms of higher-order meta-representational mentalizing mediated by the mPFC, subserving, for example, perspective taking when others are thought to be markedly different from oneself (143). Therefore, we concur with Hein and Monk (43), who interpret the maltreatment-related increase in pSTS activation while viewing threatening faces in terms of more rapid (and potentially biased) detection of others' threatening states of mind (e.g., hostile attribution bias), potentially enabling maltreated children to more efficiently navigate socially dangerous or harmful environments—a pattern we would associate more strongly with the hyper-arousal phenotype.

### Potential Alterations in Further Brain Regions

In the previous theoretical examination, we focused on four neural systems which are central for inter-individual differences in NAMA. However, there are also other brain regions that could convey differential effects based on early adverse child–caregiver interactions. One such region is the corpus callosum, the white matter structure that connects the brain hemispheres. In both neglected and abused individuals, the reduced integrity and area of the corpus callosum is a well-replicated finding (144). Teicher et al. (70) argue that these alterations might indicate an (at first) adaptive mechanism by which the affected individuals adjust to an enduring approach–avoidance conflict in the relationship to a maltreating caregiver. This notion is supported by research providing evidence for more lateralized and less integrated brain activity in maltreated individuals (145), which could be the functional correlate of reduced callosal integrity.

These functional alterations, in turn, could also underlie the “black and white” thinking as well as “splitting” characteristic of borderline personality disorder, a mental disorder that is often preceded by childhood maltreatment (146) and associated with disorganized attachment (147) and unresolved psychological trauma, as indexed by the Adult Attachment Interview (AAI) (148). Moreover, disorganized attachment has also been associated with the emergence of “segregated systems,” a regulatory strategy that entails a diminished integration of affects, expectations, and so on to prevent the individual from feeling overwhelmed in the present but resulting in continuation of mismatched or incompatible fears in the future (43). Therefore, disorganized attachment due to neglect or abuse could also be associated with the reduced integrity or area of the corpus callosum.

### Summary

We have offered above a brief overview of the *direct* and *indirect* (i.e., maltreatment-related) evidence in support of the distinction between hyper- and hypo-arousal phenotypes of disorganized attachment (summarized in Figure 3). Our proposal receives most direct support in the case of the aversion module where the caregiver primarily serves as a threatening or insufficient source of co-regulation predisposing to hyper- and hypo-arousal profiles, respectively. However, as far as alterations in the approach, emotion regulation, and mentalization modules are concerned, our suggestions remain preliminary and in need of further exploration and confirmation in light of the paucity of direct evidence. In sum, we would like to encourage future research to formulate hypotheses and examine inter-individual differences associated with disorganized attachment regarding regions of interest not only within the proposed four neural modules of NAMA but also within other brain areas implicated in early adverse child–caregiver interactions.

## Discussion

We would like to wrap up by reiterating that, unlike most prominent models in the field (67–70), we are not emphasizing alterations in developmental neurobiology across the modules of NAMA as a function of the direct impact of adverse experiences *per se*. We rather contend that the influence of adverse experiences is filtered through the child's self- and co-regulatory efforts with their caregivers. The important implication is that singular maltreatment events in an otherwise nurturing and secure attachment relationship or early adverse events occurring outside the (current) family context should have a much weaker long-term influence in our model relative to these other models (6).

However, the flip-side of this argument is that children are most vulnerable to the occurrence of persistent adversity that occurs within their primary attachment relationships, in particular, before adolescence (69). Here we have proposed the presence of neurobiologically distinct hyper- and hypo-arousal phenotypes prototypically (but not exclusively) emanating from environments characterized by caregiver-related abuse and neglect, respectively. While much *direct* evidence initially accrued in support of a hyper-arousal pattern for disorganized infants (especially regarding cortisol), recent (primarily *indirect*) evidence from severely deprived and neglected samples has increasingly begun to document an opposing hypo-arousal pattern. Furthermore, the latter group also appears to show abnormally low levels of approach- and reward-related neural activity, which may, potentially, serve as a neural substrate for the apparent lack of motivation for interpersonal co-regulation, reflecting an early need that remained largely unmet across childhood.

Our argument, inevitably, raises the issue of adequate characterization of adverse experience. Unfortunately, much neuroimaging work to date has relied on samples with highly heterogeneous and inadequately characterized child caregiving histories. A prominent case in point is that of previously institutionalized samples that are often subsumed under the umbrella term “deprivation” when typically it is very challenging to retrieve information on experiences prior to or during institutionalization. Moreover, the disruption often associated with international adoption and the abrupt shift to (typically) very caring interactions that facilitate catch-up can become sidelined. While this work is ideal for understanding sensitive windows, it is often limited in terms of dissecting differential effects of specific environments because typically too little information on the exact nature of the environments is available, though exceptions exist with considerable effort spent on characterizing the (pre-)institutional (caregiving) environment up to its direct observation [e.g., (149)]. Thus, aside from within-group analyses considering length of institutionalization, extracting more specific dose–response effects of certain attachment-specific environments is exceedingly difficult.

Another issue implicit in our model that deserves more attention in future research is variation within healthy and non-maltreated samples in terms of secure *vs*. insecure (as well as organized and disorganized) attachment^4^. Very little or no research has attempted to take this variation in the “control” group into account when deriving the specific neurobiological sequelae of adversity. What are the distinct patterns of biological measures and neural activity, anatomy and connectivity as compared to these more burdened yet nevertheless normative samples? Actually, a debate within the attachment field that is still ongoing and began with classic attachment theorists, including Main and Ainsworth, implied that disorganization is continuous with the insecure strategies (150).

It is also worth noting that our model is primarily informed by studies relying on Hesse and Main (64) conceptualization of disorganized attachment. Notably, however, an important fMRI study by Strathearn et al. (151) using Crittenden's AAI coding system detected a diminished approach system activation among mothers with increased avoidance (type A) while viewing their own *vs*. other baby's face displaying positive and negative affect^5^. Though this pattern is in keeping with our predictions regarding the hypo-arousal subtype and the Crittenden coding system may have more clinical utility (148), it is important to note that the sample in this study was composed of mothers drawn from the general population. Therefore, it is difficult to judge the extent to which such findings are more applicable to NAMA (with its focus on organized attachment) or NAMDA (with its clinical focus on attachment disruption and disorganization). As noted above, Crittenden's conceptualization of the sequelae of maltreatment or abuse from caregivers holds that children's attachment becomes markedly organized (35, 36). Notably, Crittenden's and Main's attachment categories show a poor empirical overlap (36), cautioning scholars against considering them equivalent. However, Crittenden's system also emphasizes diversity and complexity within the attachment of maltreated children (37, 152), which is consistent with the heterogeneity that we are positing here, and therefore future examination of the extent to which this system conforms to the NAMDA model may be warranted.

One further complicating factor is the question of what happens to disorganization over time. This gets at the complex issue of normative trajectories of brain development (involving proliferation, pruning, etc.) and acceleration or deceleration of brain development due to adversity (69). This cannot be addressed at great length here, but timing of assessment, onset, recency, and chronicity of adversity may be crucial determinants of structural and functional brain alterations and other neurobiological indices. This is a fundamental issue because of well-supported theories that trauma initially leads to up-regulation followed by down-regulation below the initial set-point, resulting in under-responsiveness/blunting of the stress response in the long term (153, 154).

Finally, as mentioned briefly in the introduction when describing NAMA, a paradigm shift is currently underway in social neuroscience emphasizing the assessment of two (or) more directly interacting individuals (i.e., second-person social neuroscience). In the context of attachment, this means that new research is emerging on bio-behavioral synchrony and its association with inter-individual differences in relationship quality, particularly parent–child attachment. Although recent data on organized secure vs. insecure attachment appears promising, more research is necessary to replicate and extend these novel patterns. We are not aware of any direct evidence for effects of attachment disruption and/or disorganization in second-person social neuroscience investigations. However, the first *indirect* evidence on maltreated preschoolers dovetails with our proposal, revealing a positive concordance in parasympathetic activity for abusive, but no concordance for neglectful, mother–child dyads during puzzle tasks (155). It thus remains to be seen whether the proposed dissociation between a hypo-arousal phenotype vs. a hyper-arousal phenotype also extends to patterns of bio-behavioral synchrony among disorganized dyads and, if yes, what the implication of such dissociation may be.

In closing, our focus on co-regulation in the attachment relationship as opposed to the direct impact of early adverse childhood experiences carries important implications for intervention. Thus, to the extent that disorganized attachment is part of a fundamental interpersonal risk mechanism that is self-perpetuating in the sense that it confers deficits in forming and maintaining new relationships, this deserves to be the central focus of intervention (60). Moreover, to the extent that hyper- and hypo-arousal phenotypes can emerge in the wake of early adversity, they may call for differential intervention foci. For example, children exposed to an inaccessible or insufficient source of co-regulation may benefit most from targeting the child's ability to express and the parent's capacity to perceive the child's emotional needs, helping children regain confidence in “being heard.” By contrast, in the case of a threatening source of co-regulation, it is crucial to enable children to regain a feeling of emotional and physical safety by providing corrective therapeutic experiences and focusing on the origin and meaning of frightening behaviors for caregivers and children. Analogous to foster care intervention, a central goal may be to establish new trusting relationships by enhancing understanding of children's dysregulated behavior, addressing the caregivers own attachment-related histories, and raising awareness of possibly (often subtle) threatening behaviors (156–158).

It is our hope that our extension of the NAMA to a neuroanatomical model of disrupted attachment (NAMDA) will stimulate further research and debate in the field. With the more widespread availability of advanced biological and neuroimaging techniques, the NAMDA may offer a helpful guide for organizing emerging patterns of data in the field. In turn, this may ultimately help to further advance theory and research on attachment and childhood adversity within the twenty first century and serve as point of departure for the formulation of individualized prevention and intervention strategies.

## Author Contributions

LW, CS, and PV drafted the initial manuscript. MS, MK, JK, and JB edited and made substantial comments and suggestions for revision of the manuscript. All authors made substantial contributions to the conception/ design/ interpretation of data for the work, help draft and/or revised it critically for important intellectual content and gave final approval of the version to be published as well as agreement to be accountable for all aspects of the work in ensuring that questions related to the accuracy or integrity of any part of the work are appropriately investigated and resolved.

## Conflict of Interest

The authors declare that the research was conducted in the absence of any commercial or financial relationships that could be construed as a potential conflict of interest.

## Footnotes

^1^Main's and Crittenden's views are not necessarily mutually exclusive. Thus, fear of the AF can have a disorganizing influence on behavior, especially when the child's own resources are overwhelmed and the caregiver is the only source of comfort available, as may often be the case in the SSP (fright without solution). However, fear of the AF in the same child can also have a highly organizing influence when it comes to compulsively complying with the caregiver's demands in other contexts (e.g., at home) in order to prevent the caregiver from becoming a source of fear in the first place (to which there would be no solution). Thus, fear of the AF can have a disorganizing or organizing influence for the child depending on the context in which it occurs [cf. (37) for evidence and arguments that partly support this line of argument].

^2^An apparent exception to the elevated rates in clinical samples is adult depression, i.e., neither infant disorganization is elevated among depressed mothers nor is unresolved–disorganized status elevated among adults with depression (4, 46, 47). Regarding child depression, while studies assessing attachment in infancy yield a mixed picture, those with post-infancy assessments seem to have established a reliable link between disorganization and depression (6). That said, links to disorganization seem more consistent when considering more serious forms of depression in need of treatment (4), potentially calling for subtype-specific analyses.

^3^Although Main and Solomon (33) published their seminal chapter introducing disorganized attachment in the following year, Crittenden and Ainsworth (66) evidently already had access to it and referenced this chapter.

^4^In a similar vein, another issue worthy of examination is whether individuals' secondary attachment strategies matter in terms of whether they coincide with hypo- vs. hyper-arousal, that is, if a disorganized child is disorganized/dismissing vs. disorganized/preoccupied will this lead to differential prediction regarding their patterns of neural and physiological activation? These questions are admittedly difficult to examine as they require large samples of children classified with disorganized attachment. However, the use of factor analytically derived scores of preoccupation, dismissal, and disorganization could help researchers gain statistical power to answer some of these research questions.

^5^Please note that Crittenden's coding system, in line with her theoretical perspective, does not include a disorganized category, rendering it conceivable that the findings of Strathearn et al. (151) were driven by what would be classified as higher levels of attachment disruption or disorganization within the Main classification system among what are classified as type A individuals in the Crittenden system.
